# Maclurin Exhibits Antioxidant and Anti-Tyrosinase Activities, Suppressing Melanogenesis

**DOI:** 10.3390/antiox11061164

**Published:** 2022-06-14

**Authors:** Kyoung Mi Moon, Ju-Hye Yang, Min-Kyeong Lee, Eun-Bin Kwon, Jiwon Baek, Taehyeok Hwang, Jae-Il Kim, Bonggi Lee

**Affiliations:** 1 College of Pharmacy and Research Institute of Pharmaceutical Sciences, Gyeongsang National University, Jinju 52828, Korea; omkksm@nate.com (K.M.M.); hth4822@gmail.com (T.H.); 2 Korean Medicine (KM)-Application Center, Korea Institute of Oriental Medicine (KIOM), 70 Cheomdan-ro, Dong-gu, Daegu 41062, Korea; jjuhye@kiom.re.kr (J.-H.Y.); wrld2931@kiom.re.kr (E.-B.K.); 3 Department of Food Science and Nutrition, Pukyong National University, Pusan 48513, Korea; 3633234@hanmail.net (M.-K.L.); loog_ood@naver.com (J.B.); jikim@pknu.ac.kr (J.-I.K.)

**Keywords:** maclurin, melanogenesis, tyrosinase, human skin

## Abstract

Maclurin is rich in some edible fruits such as *Morus alba* (white mulberry) and *Garcinia mangostana*. Although maclurin showed anti-cancer and antioxidant effects, its roles in ultraviolet (UV)-induced melanogenesis have not been studied. Here, we investigated the effects of maclurin in melanogenesis using skin cells and a three-dimensional human skin model. When the cytotoxicity of maclurin was examined in B16F10 cells, no cytotoxicity was found up to 20 μM. Maclurin suppressed UVB-mediated tyrosinase activation and melanin accumulation in B16F10 cells without changes in mRNA levels of melanogenesis-related genes including tyrosinase, TRP1, TRP2, CREB, and MITF. Moreover, maclurin reduced melanin contents in melan-a cells, a cell line for normal melanocytes. When applied to a human skin model consisting of the epidermis and melanocytes, maclurin significantly reduced UVB-induced melanin accumulation (~47%) in a concentration-dependent manner based on microscopic observation and Fontana-Masson staining. Protein–ligand docking simulation followed by binding residue analysis showed that maclurin may bind to inactivate tyrosinase by forming multiple hydrogen bonds and hydrophobic and aromatic interactions with the residues of tyrosinase. Together, our study suggests that maclurin may be applied as an anti-melanogenic agent.

## 1. Introduction

Although skin aging is a natural and inescapable process due to continuous exposure to deleterious environmental factors containing ultraviolet (UV), pollutants, and micro dust, the skin is an essential protective barrier against the environmental factors. Therefore, delaying skin aging has got a special interest in the field of anti-aging research. Especially, UV irradiation can result in damage to connective tissue, immune cells, and skin cells [1,2] and chronic UV exposure may ultimately induce photoaging and skin cancers by generating a substantial amount of oxidative stress [3,4]. Thus, various in vitro and in vivo studies have been performed to find dietary factors or phytochemicals to elevate intracellular antioxidant defense systems or directly scavenge reactive oxygen species (ROS) to delay skin aging.

Of several characteristics, excessive melanin accumulation is a feature of photoaging. Although melanin synthesis is a defense mechanism underlying UV-mediated skin damage [5], chronic and repeated UV exposure promotes premature aging of the skin [5,6]. Moreover, excessive melanin synthesis is closely related to pigmentation disorders such as melasma, freckles, and solar lentigo [7,8]. Melanocytes are located in the basal layer of the epidermis and secrete melanin through dendrites. Tyrosinase is an essential enzyme for melanogenesis in mammals [9], which catalyzes two rate-limiting steps in melanogenesis: the hydroxylation of tyrosine to 3,4-dihydroxyphenylalanine (DOPA) and the oxidation of DOPA to DOPAquinone [6]. Thus, tyrosinase is one of the efficient targets to suppress pigmentation disorders and stimulate skin-whitening effects.

UV-induced oxidative stress can lead to hyperpigmentation [10] although more studies are necessary to reveal precise mechanisms. A potential mechanism includes ROS-mediated nuclear translocation of microphthalmia-associated transcription factor (MITF), a transcription factor that induces melanogenesis [11]. Therefore, a decrease in oxidative stress is necessary for preventing UV-induced skin damage. In our previous study, we screened xanthone-related natural compounds for their anti-polyphenol oxidase activity and maclurin was selected as a strong antioxidant and polyphenol oxidase inhibitor. When applied to potatoes, maclurin inhibited enzymatic browning in the long term (5 weeks) [12]. Here, we applied maclurin, a major compound in mulberry (*Morus alba* L.) twigs and root bark [13], for B16F10 cells, melan-a cells, and a human skin model to investigate its anti-melanogenic effects and underlying molecular mechanisms.

## 2. Materials and Methods

### 2.1. Cell Culture

Mouse skin B16F10 melanoma cells were purchased from the Korean Cell Line Bank (Seoul, Korea). A melan-a mouse melanocyte cell line and O-tetradecanoyl phorbol-13-acetate (TPA) were purchased from Sigma-Aldrich (St. Louis, MO, USA). B16F10 cells were cultured at 37 °C in DMEM (Dulbecco’s modified Eagle; Hyclone Laboratories, Logan, UT, USA) medium including 2 mM L-glutamine, 100 units/mL penicillin, 100 μg/mL streptomycin, and 10% heat-inactivated fetal bovine serum (FBS, Hylone Laboratory). Cells were incubated in a 5% CO_2_/95% atmosphere at 37 °C. The cells were pretreated with various concentrations (indicated in the figures or figure legends) of maclurin for 24 h and then exposed to 8 mJ or 30 mJ of UVB for B16F10 cells. For UVB exposure, the cells were irradiated once from a distance of 20 cm^2^ using a VL-115.M (UV Lamp 15 W, Vilber Lourmat, Paris, France). We performed pre-experiments to check the best UVB exposure condition depending on cell models. We choose a UVB exposure condition that induces maximal changes in gene expression related to skin aging including genes related to melanogenesis, inflammation, and oxidative stress. Melan-a cells were cultured in RPMI 1640 medium supplemented with 10% FBS, 100 units/mL penicillin, 100 μg/mL streptomycin, and 200 nM TPA in 5% CO_2_/95% atmosphere at 37 °C.

### 2.2. Cell Cytotoxicity Assay

We analyzed cell cytotoxicity using the EZ-Cytox kit (Enhanced cell viability assay kit). The kit can measure cell viability with the principle of which colored spores are formed by mitochondrial NADH-dehydrogenase activity in living cells. First, B16F10 cells were incubated in 96-well plates at a density of 1 × 10^4^ cells/well at 37 °C in a 5% CO_2_ incubator for 24 h. B16F10 cells were treated with maclurin at various concentrations (10–30 μM). After 1 day, 10 μL of EZ-Cytox reagent was added to each well. After 2 h, it was shaken for 1 min before measuring absorbance and measured at 450 nm using a plate reader.

### 2.3. Melanin Contents

B16F10 cells were incubated in 6-well plates at a density of 5 × 10^4^ cells/well at 37 °C in a 5% CO_2_ incubator for 24 h. B16F10 cells were pretreated with maclurin at various concentrations (2–15 μM) for 1 h before it was stimulated to UVB radiation for 3–4 days. The cells are washed with PBS and dissolved with 1 N NaOH and reacted to 60 °C for 1 h. The melanin contents level was measured at 490 nm using a microplate reader. To measure melanin contents in melan-a cells, a previously described method was used [14].

### 2.4. In Vitro Tyrosinase Inhibition Assay

Tyrosinase inhibition assay was measured by using L-tyrosine as a substrate and the tyrosinase purified from mushrooms. Briefly, 10 μL of maclurin and 170 μL of 50 mM phosphate buffer including NaH_2_PO_4_-NaHPO_4_ (pH 6.8) were added to the 96-well plates. Next, 20 μL of tyrosinase (500 units) were treated on each well and incubated at 37 °C for 30 min. The absorbance was measured at 490 nm using a microplate reader.

### 2.5. In Vitro ROS and ONOO—Scavenging Activity

The ROS or ONOO—scavenging activity was measured by fluorescence probe which was 2′,7′-dichlorodihydrofluorescein diacetate (DCFDA) or oxidation of dihydrorhodamine (DHR123), respectively. Briefly, to determine the level of ROS, 10 μL of maclurin was mixed with 190 μL of 50 mM phosphate buffer, and then 50 μL of 2′,7′-dichlorofluorescin (DCF) which was made using dichlorodihydrofluorescein diacetate (DCFDA) and esterase was added to determine ROS. To determine the level of ONOO^−^, rhodamine solution was prepared by using 50 mM sodium phosphate buffer, 90 mM NaCl, 5 mM diethylene-triamine pentaacetate [DTPA], and DHR 123. About 10 μL of maclurin was added to the rhodamine solution for a final volume of 200 μL. The ROS or ONOO^−^ scavenging activity was determined by measuring the fluorescence intensity change every 5 min for 30 min using a fluorescence plate reader (SpectraMax i3, Molecular Devices, San Jose, CA, USA) at excitation of 485 nm and emission of 530 nm.

### 2.6. 3D Artificial Human Skin

3D artificial human skin models (Neoderm-ME) were purchased from the Tegoscience company (Seoul, South Korea). When culturing artificial skin, a maintenance medium which is provided by Tego Science containing 10% FBS was used. It was used to evaluate the inhibitory effect of maclurin on skin aging. The 3D artificial human skin was pretreated with DMSO (vehicle) or maclurin for 1 h before it was exposed to UVB radiation, and it was incubated for 5 days using the maintenance media provided by the company after UVB exposure. To investigate the accumulation of melanosomes, microscopic analysis was conducted from 1 day to 5 days, and the images which were obtained by microscopic analysis were analyzed by the Image J software program, which is to semi-quantify the darkening of the skin. To visualize intracellular melanin formation and collagen degradation, Fontana-Masson Staining and Masson’s Trichrome staining were performed in the Neoderm-ME model, respectively. The 3D artificial human skin samples were fixed in 4% paraformaldehyde overnight at room temperature and the 3D human skin sample were analyzed by Tegoscience Company (Seoul, Korea).

### 2.7. In Silico Protein–Ligand Docking Simulation

To perform a Docking simulation of tyrosinase and maclurin, the crystal structures of human tyrosinase were obtained from the Protein Data Bank (PDB ID: 5I38) and 3D structures of maclurin were obtained from the PubChem site (https://pubchem.ncbi.nlm.nih.gov, accessed on 1 May 2021). Docking simulations were analyzed between the tyrosinase and maclurin by using the AutoDock 4.2 program offering automated docking capabilities. Moreover, pharmacophore analysis was evaluated from LigandScout 3.1 program to predict the possibility of how structurally maclurin could bind to a tyrosinase.

### 2.8. Measurement of Tyrosinase Activity in B16F10 Cells

B16F10 melanoma cells (5 × 10^4^ cells/well) were dissolved in 100 µL of a 50 mM sodium phosphate buffer (pH 6.5) containing 1% TritonX-100 and 0.1 mM phenylmethyl sulfonyl fluoride (PMSF). The sample was frozen at −80 °C for 30 min, and the supernatant was centrifuged at 12,000× *g* for 30 min at 4 °C. The 80 μL of supernatant solution and 20 μL of L-DOPA (2 mg/mL) were added to the 96-well plate well. The sample was measured with absorbance every 10 min at 492 nm for an hour at 37 °C.

### 2.9. Statistical Analysis

All experiments were performed three times. The data are shown as mean ± standard error of the mean (SEM). Different groups were compared by using a one-way analysis of variance (ANOVA) followed by Dunnett’s multiple comparison test as post-analysis. In all data, a probability value of *p* values < 0.05 was used as standard statistically significant.

## 3. Results

### 3.1. Tyrosinase Inhibition and ROS Scavenging Activities of Maclurin

**Figure 1:**
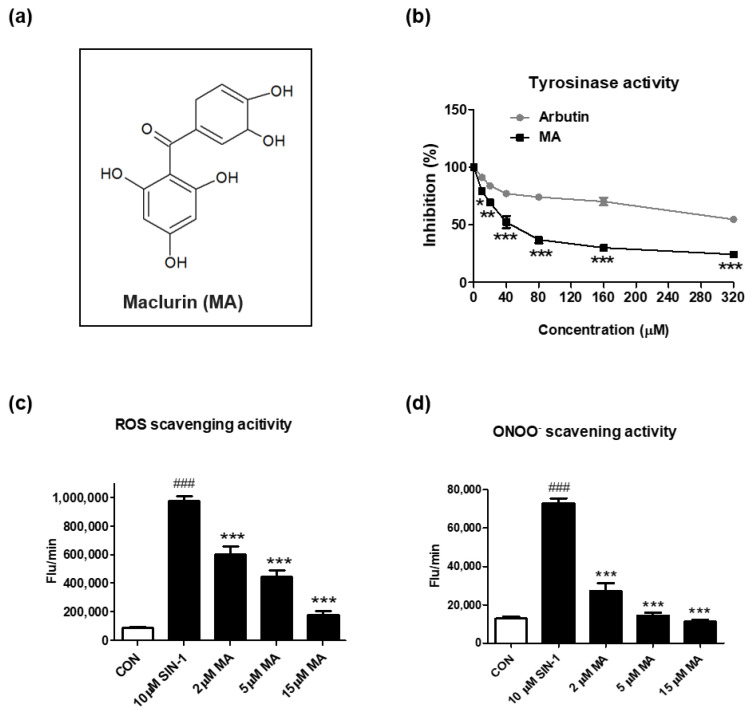
Inhibitory effects of maclurin on tyrosinase and oxidative stress in cell-free systems. (**a**) The chemical structure of maclurin. (**b**) The inhibitory effects of maclurin and arbutin on tyrosinase were measured in the cell-free system using L-tyrosine as the subtract and tyrosinase as the enzyme. To examine the inhibitory effect of maclurin on oxidative stress, reactive oxygen species (ROS) and peroxynitrite (ONOO^−^) were generated by 3-morpholinosydnonimine (SIN-1). (**c**) ROS and (**d**) ONOO^−^ were analyzed by DCFDA and DHR123 as fluorescent probes in a cell-free system after 30 min incubation. Results represented the mean value ± SEM at least three times. The data were compared by ANOVA, followed by Dunnett’s multiple comparison test. *^###^ p* < 0.001 compared with the non-treated control group and * p < 0.05, ** p < 0.01, *** p < 0.001 compared to the SIN-1-treated group or the arbutin-treated group.

Maclurin, a yellowish phenolic compound, is a xanthone precursor molecule (Figure 1a) found in *Morus alba* (white mulberry), mulberry twigs, and *Garcinia mangostana* [13,15]. To investigate the inhibitory effects of maclurin on skin aging, we measured tyrosinase inhibitory and ROS scavenging activities of maclurin in a cell-free system. When compared to arbutin, a well-known whitening agent, maclurin exhibited stronger inhibitory activity against tyrosinase (Figure 1b). To test antioxidant activity, 3-morpholinosydnonimine (SIN-1), a strong inducer for ROS and ONOO^−^, was used to increase oxidative stress. Maclurin treatment at 2–15 µM significantly suppressed the levels of ROS and ONOO^−^ in a concentration-dependent manner (Figure 1c,d).

### 3.2. Maclurin Suppressed Melanin Levels in Melanocyte Cell Models

**Figure 2:**
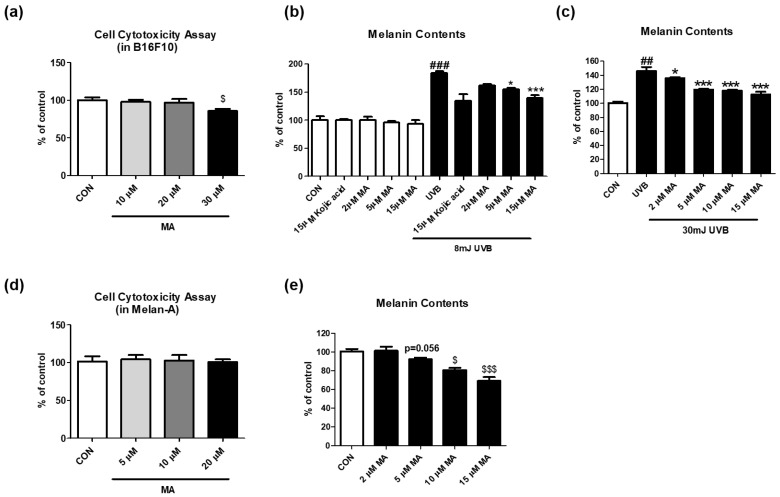
Effect of maclurin on melanogenesis in B16F10 cells. (**a**) Cell cytotoxicity was measured in B16F10. The cells were treated with various concentrations (10, 20, and 30 μM) of maclurin for 24 h. The cell viability was evaluated by treatment of cell toxicity solution based on materials and methods. The effects of maclurin on spontaneous melanogenesis were investigated using B16F10 cells without any extrinsic stimulations. To test the melanin inhibitory effect of maclurin against UVB exposure, the cell was pretreated with maclurin for 1 hr and then exposed to (**b**) 8 mJ or (**c**) 30 mJ of UVB radiation followed by incubation for 3 days. To investigate the anti-melanogenic effects of maclurin in normal melanocytes, melan-a cells were cultured using a method indicated in the Section 2, (**d**) cell cytotoxicity and (**e**) melanin contents were measured. All data represented the mean ± SEM at least three times. ^$^ *p* < 0.05 and ^$$$^ *p* < 0.001 compared to the control group. *^##^ p* < 0.01, *^###^ p* < 0.001 compared with the non-exposed control group and * p < 0.05 and *** p < 0.001 compared to the UVB-exposed group.

Based on the data of the cell-free experiments, we further tested whether maclurin affects cell cytotoxicity in multiple skin cell types including B16F10 (mouse melanoma), and also examined whether maclurin inhibits UVB-induced melanogenesis using B16F10 cells. Maclurin exhibited no cytotoxicity in these cell lines at 10–20 µM (Figure 2a). We studied its effect on melanin accumulation using B16F10 cells pre-treated with kojic acid, a well-known whitening agent, or maclurin followed by UVB exposure at different strengths. Without any stimulations, maclurin did not affect melanin accumulation at 2–15 μM (Figure 2b). On the other hand, UVB exposure at 8 mJ highly elevated melanin levels in the cells, and maclurin decreased it post UVB exposure (Figure 2b). When maclurin (2–15 µM) was applied in B16F10 cells exposed to UVB at 30 mJ, melanogenesis was reduced in a concentration-dependent manner (Figure 2c). Because the B16F10 cell line is derived from the skin tissue of a mouse with melanoma, we used a normal mouse melanocyte cell line (melan-a) that shows a similar phenotype as the primary mouse melanocytes [16]. Consistent with the previous results using B16F10 cells, maclurin at the concentration that did not show cytotoxicity (Figure 2d) significantly reduced melanin content in a concentration-dependent manner in melan-a cells (Figure 2e).

### 3.3. Maclurin Suppressed Cellular Tyrosinase Activity without Altering mRNA Levels of Genes Related to Melanogenesis

**Figure 3:**
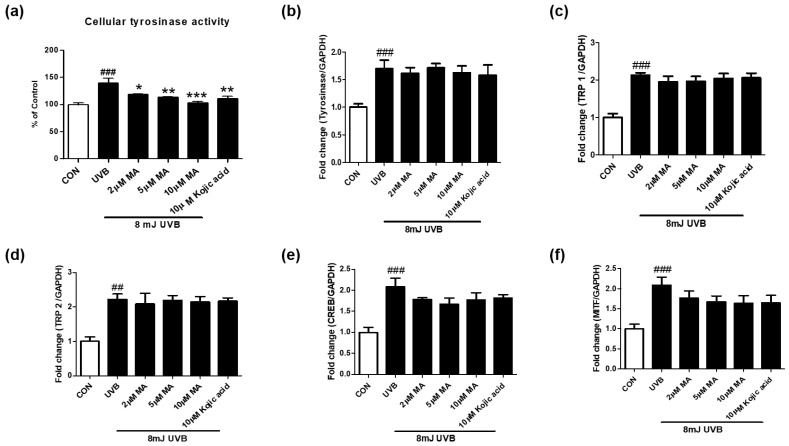
Maclurin suppressed cellular tyrosinase activity without altering mRNA levels of genes related to melanogenesis. (**a**) Cellular tyrosinase activity was measured using B16F10 cells after the pretreatment of maclurin for 1 h followed by UVB exposure (8 mJ). QPCR was performed to measure mRNA levels of (**b**) tyrosinase, (**c**) TRP1, (**d**) TRP2, (**e**) CREB, and (**f**) MITF. All data represented the mean ± SEM at least three times. ^##^ *p* < 0.01, ^###^ *p* < 0.001 compared with the non-exposed control group and * *p* < 0.05, ** *p* < 0.01, and *** *p* < 0.001 compared to the UVB-exposed group.

We tested whether maclurin regulates cellular tyrosinase activity and mRNA levels of genes related to melanogenesis in UVB-exposed B16F10 cells. Maclurin treatment (2–10 µM) significantly decreased the activity of cellular tyrosinase post UVB exposure (8 mJ) (Figure 3a). Although UVB exposure significantly elevated mRNA levels of genes related to melanogenesis, MITF, cAMP-response element-binding protein (CREB), tyrosinase, tyrosinase-related protein (TRP1), and TRP2, maclurin treatment did not alter the mRNA levels of these genes (Figure 3b–f). These data suggest that maclurin inhibits UVB-induced melanin accumulation partly via inhibiting cellular tyrosinase activity.

### 3.4. Maclurin Suppressed Melanin Levels in a Human Skin Model

**Figure 4:**
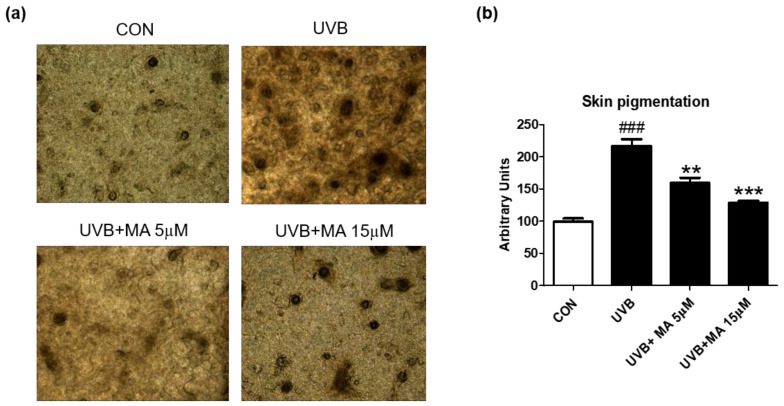
Inhibitory effect of maclurin on melanogenesis in 3D artificial human skin. The pigmented human skin model (Neoderm-ME) was pretreated with maclurin and exposed to UVB radiation, followed by incubating in the maintenance media offered by the company for 5 days. (**a**) Microscope images of 3D skin ME model, which is pretreated with DMSO or maclurin, and then exposed to UVB (10 mJ) or not. (**b**) The pigmentation of the skin was analyzed by the Image J software program. The results were represented the mean value ± SEM at least three times. All data were compared by ANOVA, followed by Dunnett’s multiple comparison test. ^###^ *p* < 0.001 compared with the control group and ** *p* < 0.01, *** *p* < 0.001 compared to the UVB-exposed group.

**Figure 5:**
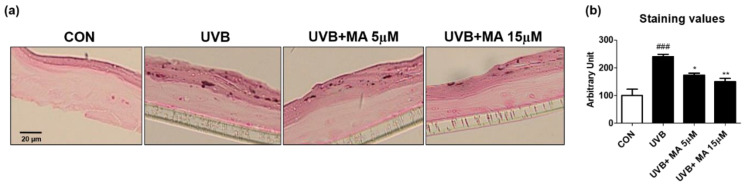
Fontana-Masson staining results in 3D artificial human skin pretreated with maclurin. The pigmented human skin model (Neoderm-ME) was pretreated with maclurin and exposed to UVB radiation, followed by incubating in the maintenance media offered by the company for 5 days. (**a**) Fontana-Masson staining images showed differences in melanosome in the epidermis of the pigmented human skin model. (**b**) Fontana-Masson staining images were analyzed by the Image J analysis software. The results were represented the mean value ± SEM at least three times. All data were compared by ANOVA, followed by Dunnett’s multiple comparison test. ^###^ *p* < 0.001 compared with the control group and * *p* < 0.05, ** *p* < 0.01 compared to the UVB-exposed group.

To verify that maclurin inhibits skin pigmentation in humans, we cultured a viable, three-dimensional, reconstituted human epidermis consisting of human melanocytes and keratinocytes. We pre-treated the human skin model with maclurin for 1 h and cultured it in the maintenance medium for additional 5 days after UVB exposure. Compared to the unexposed one, the human skin model was darkened after UVB exposure as expected and maclurin at 5 and 15 µM suppressed skin darkening in a concentration-dependent manner (Figure 4a,b). Fontana-Masson staining showed that maclurin decreased UVB-induced melanosome formation in the epidermis (Figure 5a,b), indicating that maclurin elevates a whitening effect in the human skin model.

### 3.5. Maclurin May Bind to and Deactivate Tyrosinase

**Figure 6:**
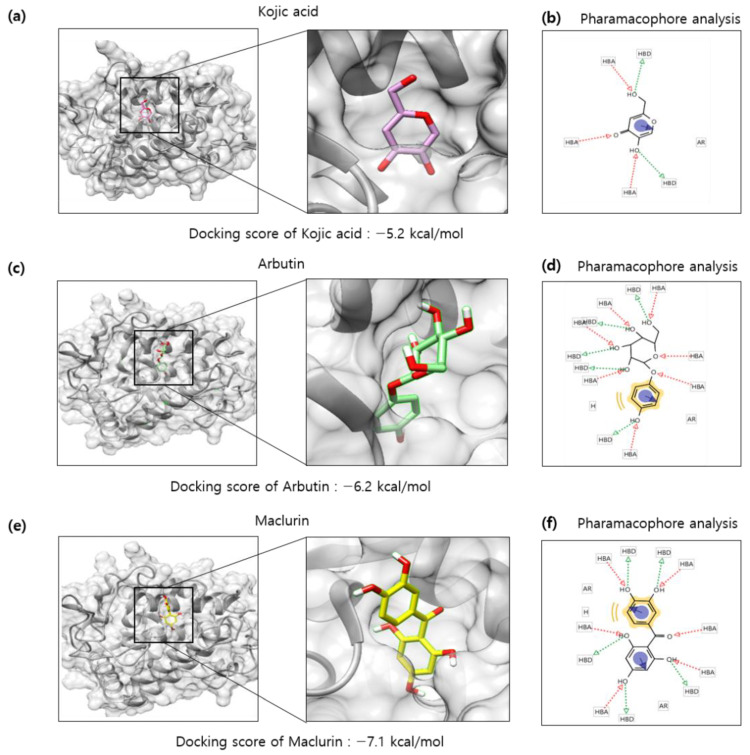
In silico docking simulation of tyrosinase and maclurin. Protein-ligand docking simulation was performed using AutoDock Vina. It was analyzed between human tyrosinase (5I38) and (**a**) kojic acid, (**c**) arbutin, and (**e**) maclurin as a receptor and ligands, respectively. In a marked square box, it represents the tyrosinase binding site with an enlarged image. The binding residues analysis of (**b**) kojic, (**d**) arbutin, or (**f**) maclurin with tyrosinase was performed by pharmacophore analysis.

Because maclurin inhibited tyrosinase activity in the cell-free experiment using mushroom tyrosinase, it is hypothesized that maclurin directly binds to and inactivates tyrosinase. To examine it, a protein–ligand docking simulation was performed using the AutoDock Vina 4.2 software. Tyrosinase (PDB 5I38) was selected to predict binding affinity between various compounds and a protein. The expected binding affinity between kojic acid and tyrosinase was −5.2 kcal/mol and the affinity between arbutin and tyrosinase was −6.2 kcal/mol (Figure 6a,c), whereas the affinity between maclurin and tyrosinase was −7.1 kcal/mol (Figure 6e), indicating that maclurin probably binds to tyrosinase with stronger affinity than arbutin and kojic acid do. To better understand how each compound binds to tyrosinase, binding mode analysis was performed using the LigandScout 3.1 software (Maria Enzersdorf, Austria, Europe). Based on the structure, kojic acid may have three hydrogen bond acceptors and two hydrogen bond donors which can be associated with tyrosinase binding and also form one aromatic interaction with tyrosinase (Figure 6b). Arbutin may have seven hydrogen bond acceptors and five hydrogen bond donors related to tyrosinase binding and also form one hydrophobic and aromatic interaction with tyrosinase (Figure 6d). On the other hand, maclurin probably has six hydrogen bond acceptors and five hydrogen bond donors associated with tyrosinase binding and also forms one hydrophobic and two aromatic interactions with tyrosinase (Figure 6f).

## 4. Discussion

Although maclurin showed anti-cancer and antioxidant effects [14,15,16], very limited studies were available about its beneficial effects on the skin. Nevertheless, a study demonstrated that synergetic effects of maclurin on ginsenoside compound K mediated the suppression of transcriptional expression of matrix metalloproteinase-1, a key factor for wrinkle formation, in the HaCaT human keratinocyte cell line [17]. Moreover, the antioxidant and antityrosinase activity of the ethanol extract of mulberry twigs were reported [13]. Further high-performance liquid chromatography analysis showed that the ethanol fraction includes maclurin, rutin, isoquercitrin, resveratrol, and morin [13]. Another study showed that maclurin ameliorated enzymatic browning for long-term and elevated antioxidant capacity when added to potato supernatant [12]. Because enzymatic browning and melanogenesis may share a common mechanism including tyrosinase activation, we tested the effects of maclurin on UVB-induced pigmentation using B16F10 melanoma cells, melan-a normal melanocytes, and three-dimensional human skin models. The pre-treatment of maclurin ameliorated UVB-induced melanogenesis presumably by direct binding to and inhibiting tyrosinase by forming multiple hydrogen bonds and aromatic and hydrophobic interactions with the residues of tyrosinase without notable changes in mRNA expression of genes related to melanogenesis, such as tyrosinase, TRP1, TRP2, CREB, and MITF. Moreover, maclurin exhibited strong antioxidant capacity based on ROS and ONOO^−^ scavenging assays. These results were consistent with other studies [12,17]. When maclurin was treated in mesenchymal stem cells after the induction of oxidative stress by hydroxyl radical (OH), maclurin appears to scavenge OH radicals possibly by metal-chelating and direct radical scavenging, thereby protecting against oxidative damage to DNA and mesenchymal stem cells [17]. Another study indicated that maclurin addition to potato extract elevated antioxidant capacity and the effect was greater than that of vitamin C [12]. Antioxidants can react with oxygen and thereby inhibiting the initiation of enzymatic browning. They can also react with the intermediate products, thus breaking the chain reaction and inhibiting melanin formation [17]. Based on these observations, we assume that maclurin exerts anti-melanogenic activity partly through elevating antioxidant capacity and direct binding to and inactivating tyrosinase.

Although docking simulation exhibited that maclurin may inactivate tyrosinase based on the chemical structure to bind to the active site of tyrosinase, there may be more mechanisms underlying maclurin-mediated skin whitening effects due to the strong antioxidant characteristics evidenced by the strong scavenging effects of maclurin on ROS and ONOO^−^ in the cell-free experiments. Our previous study showed that maclurin significantly elevated antioxidant capacity when added to foods [12]. When the skin epidermis is irradiated with UV rays, ROS-mediated tyrosinase expression is induced for melanogenesis through the p53-POMC-α-MSH [1] and the mitogen-activated protein kinase (MAPK)-MITF pathways [2]. A study showed that swertiajaponin, a natural compound with antioxidant effects, inhibited oxidative stress-mediated MAPK/MITF signaling, thereby suppressing melanin accumulation in both cellular and human skin models [5]. In our study, maclurin-mediated antioxidant effects may likely down-regulate these signaling pathways although it needs to be confirmed by in vitro experiments in the future.

The importance of intracellular antioxidant genes on skin whitening effects was also shown in in vitro gain-of-function studies. FoxO3 and FoxO6 are antioxidant genes that suppress oxidative stress by up-regulating intracellular antioxidant genes including SOD and catalase [3,4]. When FoxO3 or FoxO6 was knocked down in UV-irradiated B16F10 cells, melanin was significantly increased, whereas this effect was reversed by FoxO3 or FoxO6 activation. These data indicate that not only antioxidant capacity by direct scavenging of oxidative stress inducers but also intracellular antioxidant capacity is important to inhibit melanin production. Although it is not tested in the current study, the effects of maclurin on intracellular antioxidant genes including FoxOs, SOD, catalase, etc., will be necessary to further reveal the mechanisms underlying its anti-melanogenic effects.

It needs to be pointed out that data from docking simulations have intrinsic limitations. One of the major limitations is the lack of confidence in the ability of scoring functions to suggest precise binding energies. It arises from that some of intermolecular interaction terms are hard to predict precisely, such as solvation effect and entropy change [18]. Moreover, some intermolecular interactions are not considered in scoring functions which have been proven to be of significance. Isothermal titration may be needed to prove actual reactions between maclurin and tyrosinase including binding affinity, stoichiometry, entropy, and enthalpy of the binding reaction.

On the other hand, the different effects of maclurin on melanogenesis have also been reported in a recent study [19]. Maclurin elevated cellular tyrosinase activity as well as cellular melanin levels in human melanocytes [19]. The melanogenic capacity of maclurin depends on up-regulated MITF gene expression, which is probably induced by the activation of both p38 MAPK/CREB and cAMP/PKA/CREB signaling [19]. In this study, human epidermal melanocytes were used with no treatment of intrinsic or extrinsic melanogenic inducers such as UV. On the other hand, we used B16F10 melanoma cells and a three-dimensional human epidermis consisting of human melanocytes and keratinocytes with the treatment of UVB which is a strong inducer for melanogenesis at least partly by elevating oxidative stress, inflammation, and α-MSH signaling pathways. Thus, huge differences between the two studies are that one study examined the effects of maclurin on spontaneous melanogenesis [19] and our study tested the effects of maclurin on UVB-stimulated melanogenesis. Because UVB stimulates multiple melanogenic signaling pathways including oxidative stress and MC1R signaling that elevate tyrosinase activity [4,20], the anti-melanogenic effects of maclurin in our study can be derived from its strong antioxidant and tyrosinase-inhibitory activities possibly by direct scavenging ROS and binding to and inactivating tyrosinase. This result is consistent with a previous study showing the inhibitory activity of maclurin on mushroom-derived tyrosinase and the darkening of potato supernatant [12]. Furthermore, our data showed that maclurin treatment in B16F10 cells without any stimulations did not affect melanin accumulation, further supporting that maclurin is only effective in UVB-induced melanogenesis in our experimental conditions.

Safety is an important issue to be considered in the pharmaceutical and cosmetic industry to apply a certain compound as a drug or cosmetics. Although it has been reported that maclurin is rich in some edible fruits such as *Morus alba* (white mulberry) and *Garcinia mangostana* [13,15], the safety of maclurin application as a drug or cosmetics is not widely examined. Nevertheless, a study showed that maclurin up to 70 µM did not exhibit cellular toxicity in HaCaT human keratinocytes, but it slightly reduced cell viability at 100 µM [21]. Another study showed that maclurin treatment (38.2–191.1 µM) protected mesenchymal stem cells from hydroxyl radical-induced cell cytotoxicity [17]. In the current study, maclurin showed slight toxicity at 30 µM in the B16F10 cell line. Future studies are necessary to test the short-term and long-term side effects of maclurin in various animal models depending on the purpose of use.

## 5. Conclusions

Maclurin efficiently suppressed UVB-mediated skin pigmentation in mouse-derived melanocytes and three-dimensional, reconstituted human skin models. The mechanisms include but are not limited to the antioxidant capacity and inactivation of tyrosinase in melanocytes. However, further experiments will be necessary using human-derived melanocytes to confirm the anti-melanogenic effects in humans. Nevertheless, maclurin may be applied as an anti-skin aging agent.

## Data Availability

Data is contained within the article.
